# Post-Traumatic Stress, Workplace Violence, Resilience, and Burnout: A Path Analysis Among Korean Paramedics

**DOI:** 10.3390/healthcare13192519

**Published:** 2025-10-04

**Authors:** Jieun Sung, Nayoon Lee

**Affiliations:** 1 Graduate School, College of Nursing, Dong-A University, 32 Daesingongwon-ro, Seo-gu, Busan 49201, Republic of Korea; dawn1012@naver.com; 2 College of Nursing, Dong-A University, 32 Daesingongwon-ro, Seo-gu, Busan 49201, Republic of Korea

**Keywords:** paramedics, burnout, post-traumatic stress, workplace violence, resilience

## Abstract

**Background/Objectives**: Paramedics frequently encounter potentially traumatic events and workplace violence, increasing their risk of burnout. Resilience may attenuate these effects. We examined the pathways through which post-traumatic stress (PTS) and workplace violence influence burnout and clarified the role of resilience among Korean paramedics. **Methods:** We studied duty-related trauma and violence experienced by 208 Busan Fire Department paramedics using standardized measures of PTS, workplace violence, resilience, and burnout. Using structural equation modeling, we tested the direct and indirect effects; covariates included sex, nursing license, and intention to stay. **Results**: PTS was most strongly associated with burnout, whereas workplace violence was indirectly associated with burnout through PTS. Resilience reduced PTS, yielding an indirect protective effect on burnout; however, it had no direct effect on burnout. Holding a nursing license and lack of intention to stay were significantly associated with burnout, and female sex and lack of intention to stay were indirectly associated with burnout via PTS. **Conclusions**: Burnout is primarily driven by PTS, and workplace violence amplifies PTS and indirectly exacerbates burnout. Strengthening violence prevention/response systems, early PTS screening/treatment, and resilience-building programs is warranted, with targeted support for vulnerable subgroups.

## 1. Introduction

### 1.1. Background

Paramedics serve as first responders at disaster scenes, and they are crucial in safeguarding lives and public safety. They frequently encounter patients experiencing cardiac arrest or severe trauma whose care requires rapid judgment and immediate decision-making, and they are repeatedly exposed to traumatic situations, such as witnessing or treating severely injured bodies at accident sites [1,2]. In addition, they often experience workplace violence (WPV), including verbal abuse and physical assault, committed not only by patients but also by caregivers and other civilians [3]. In 2018, a paramedic in Korea died after being assaulted by an intoxicated patient, highlighting workplace violence against paramedics as a serious social issue [4]. Following this incident, the Korean government enacted legislation in 2019 to strengthen the penalties for assault on paramedics [5]. However, according to the National Fire Agency’s annual statistics, 132 cases of assaults on paramedics were reported in 2014 [6], and 245 cases were reported in 2023 [6], indicating that despite heightened social attention and stronger penalties, incidents of WPV against paramedics have continued to increase. Paramedics frequently experience various mental health difficulties, including post-traumatic stress (PTS), anxiety, depression, sleep disorders, and suicidal ideation, which are closely associated with repeated exposure to trauma and violence [7,8,9].

Among the various mental health issues faced by paramedics, burnout deserves particular attention. Burnout is a psychological syndrome characterized by emotional exhaustion, depersonalization, and reduced personal accomplishment [10], and paramedics are known to experience particularly high levels this syndrome [11]. Burnout not only causes a variety of physical and psychological health problems but also negatively affects both individuals and organizations by contributing to job dissatisfaction, presenteeism, increased turnover intention, and decreased job performance quality [12,13]. Therefore, preventing burnout among paramedics, who work in emergency settings directly related to saving lives should be considered both as an issue of personal health and welfare and as a critical task to ensure the stability and sustainability of an emergency medical system (EMS).

PTS and WPV have been consistently reported as major contributors to burnout among paramedics [7,14,15]. Owing to the nature of their work, paramedics are repeatedly exposed to horrific scenes such as severe injury accidents as well as scenes involving suicide survivors and infant deaths. These traumatic experiences can trigger PTS [16]. PTS manifests through symptoms such as re-experiencing, avoidance, and hyper-arousal, which lead to difficulties in emotional regulation and reduced resilience, ultimately accelerating emotional exhaustion and serving as a key factor in burnout [17]. In addition, paramedics are known to experience various forms of WPV, including physical assault, verbal abuse, threats, and being attacked or intimidated with weapons [18]. Although several policies have been implemented to prevent WPV against paramedics, including nationwide public campaigns, the development of on-site response protocols, installation of in-ambulance CCTV, and provision of wearable cameras, WPV frequency is increasing continuously [19]. WPV lowers work efficacy and job satisfaction while heightening negative self-perceptions, thereby exacerbating burnout. Numerous studies have reported that WPV significantly affects burnout in other public-facing professions, such as nurses, social workers, and teachers [20,21].

However, not all paramedics respond similarly to trauma or experiences of violence, and resilience has gained attention as a psychological factor that can explain these differences. Resilience refers to an individual’s psychological capacity to effectively overcome stress or adversity and recover from it [22]. Previous studies conducted across various occupational groups have reported that resilience functions as a protective factor that mitigates burnout [23,24]. Therefore, identifying the role of resilience in the process through which the PTS and WPV experienced by paramedics leads to burnout has considerable practical and academic significance.

Previous studies have mainly examined the individual effects or simple correlations among traumatic experiences, WPV, resilience, and burnout [11,12,14], and limited studies have comprehensively analyzed the specific pathways through which these variables interact and lead to burnout. In particular, scarce empirical research explains how paramedics exposed to the dual burden of trauma and WPV develop burnout and how resilience functions in this process.

Against this backdrop, the present study aimed to structurally analyze the effects of WPV and PTS on burnout among paramedics and to comprehensively examine the role of resilience in this process. This approach is expected to move beyond simply identifying associations among variables, additionally suggesting practical directions for interventions and serving as a basis for resilience enhancement programs and organizational policy development.

### 1.2. Purpose

This study aimed to examine the differences in PTS, experiences of WPV, resilience, and burnout among paramedics according to their general characteristics; analyze the correlations among these variables; and construct and validate a path model of the relationships among PTS, WPV, resilience, and burnout.

## 2. Methods

### 2.1. Study Design

This cross-sectional study examined the relationships between PTS, WPV, resilience, and burnout in paramedics.

### 2.2. Participants and Data Collection

This study was conducted among paramedics engaged in emergency medical services in Busan, South Korea. The inclusion criteria were individuals aged 18 years or older who understood the study purpose and voluntarily agreed to participate. Paramedics employed at the Busan Fire and Disaster Headquarters were eligible, and participation was entirely voluntary. Only those who reviewed the information sheet and provided electronic informed consent were included. The required sample size was calculated using the G*Power 3.1.9.7 program, with a significance level of 0.05, power of 0.95, and effect size of 0.15, which yielded 189 participants. Factoring in a 10% dropout rate, 208 completed questionnaires were collected and used for the final analysis.

This study was approved by the Busan Fire and Disaster Headquarters after providing a detailed explanation of its purpose and procedures. Data were collected between 13 June and 3 July 2025, through online surveys to ensure that participation did not interfere with dispatch duties or other work responsibilities. With the cooperation of the Busan Fire and Disaster Headquarters, the survey URL and QR code were posted on an internal online bulletin board and the Busan Firefighter Psychological Support Team KakaoTalk channel. Participants accessed the survey via the URL or QR code provided and completed the questionnaire using Google Forms. The survey began with an information sheet, and only those who provided voluntary informed consent by checking the consent box were allowed to proceed. The online form was designed such that participants could not move to the next section without completing the required responses.

### 2.3. Measurements

The study’s structured online questionnaire comprised questions on participants’ general characteristics (sex, age, marital status, position, shiftwork, licenses, years of continuous service, frequency of mobilization, monthly salary, and intention to stay), along with instruments measuring PTS, WPV experience, resilience, and burnout. For all instruments, scale scores were calculated as the mean of the item responses, such that the final scores reflected the average value within the original response range of each tool.

#### 2.3.1. Post-Traumatic Stress

In this study, the Korean version of the post-traumatic stress disorder (PTSD) checklist for the DSM-5 (PCL-5-K), originally developed by Weathers et al. and adapted to Korean by Kim et al. [25], was used with permission. This tool comprises 20 items rated on a five-point Likert scale ranging from 0 (“not at all”) to 4 (“extremely”), with higher scores indicating greater severity of PTSD symptoms. The reliability of the tool in Kim et al.’s study was Cronbach’s α = 0.97, and in the present study, it was also 0.97.

#### 2.3.2. Workplace Violence

In this study, an instrument measuring the types and frequency of violence, revised and supplemented by Yun [26] based on the findings of Sohn [27], was used with permission. This instrument comprised 16 items across three subdomains: verbal violence (four items), physical threat (five items), and physical violence (seven items). The reliability of the instrument was Cronbach’s α = 0.87 in Yun’s study and 0.94 in the present study.

#### 2.3.3. Resilience

In this study, the validated Korean version of the Connor–Davidson Resilience Scale (CD-RISC) originally developed by Connor and Davidson was used with permission [28]. The instrument comprises 25 items across seven subdomains: hardiness (seven items), coping (five items), adaptability/flexibility (three items), meaning/purpose (four items), optimism (two items), regulation of perception and emotion (two items), and self-efficacy (two items). Each item is rated on a five-point Likert scale ranging from 0 (“not true at all”) to 4 (“true nearly all the time”), with higher scores indicating greater resilience. The Cronbach’s α of the instrument was 0.89 at the time of development and 0.97 in this study.

#### 2.3.4. Burnout

This study used a validated Korean version of the Maslach Burnout Inventory (MBI), with permission. The original instrument was developed by Maslach and Jackson [29]. The instrument comprises 22 items across three subdomains: emotional exhaustion (nine items), personal accomplishment (eight items), and depersonalization (five items). Each item is rated on a seven-point Likert scale ranging from 0 (“never”) to 6 (“every day”). The items in the personal accomplishment subdomain (eight items) were reverse-coded such that higher scores indicated greater burnout. The Cronbach’s α of the instrument was 0.76 at the time of development and 0.91 in this study.

### 2.4. Data Analysis

The collected data were analyzed using the SPSS (ver. 29), and AMOS (ver. 29) programs. First, descriptive statistics, including frequency, percentage, mean, and standard deviation, were calculated to examine general characteristics, PTS, WPV, resilience, and burnout. Burnout was compared across general characteristics using *t*-tests and ANOVA, with Scheffé’s test applied for post hoc analysis. The relationships among major variables were examined using Pearson’s correlation coefficients, and normality was assessed by skewness and kurtosis. Second, to investigate the relationships among PTS, WPV, resilience, and burnout, structural equation modeling (SEM) was conducted using the AMOS 29 program. Path coefficients were estimated using the maximum likelihood method. Model fit was evaluated using χ^2^, GFI, CFI, NFI, IFI, TLI, SRMR, and RMSEA. Finally, the statistical significance of indirect and total effects was tested using bootstrapping.

### 2.5. Ethical Considerations

To ensure the ethical protection of participants, this study was approved by the Dong-A University Institutional Review Board in Korea (initial approval: IRB No. 2-1040709-AB-N-01-202504-HR-020-04, 5 June 2025; amendment approval: IRB No. 2-1040709-AB-N-01-202504-HR-020-06, 30 June 2025) prior to data collection. The information sheet explained the study’s purpose and procedures, the voluntary nature of participation, the right to refuse or withdraw at any time, and the potential benefits and risks. It also stated that the collected data would be used solely for research purposes, anonymized, stored for three years after the completion of the study, and permanently deleted thereafter. Only individuals who voluntarily provided informed consent after reviewing the information sheet were allowed to complete the survey, and a small gift was provided upon survey completion.

## 3. Results

### 3.1. Burnout According to General Characteristics

The mean age of participants was 37.5 ± 7.1 years, with 80.3% (*n* = 167) being male and 69.2% (*n* = 144) married. Regarding their position, 22.6% (*n* = 47) were Firefighters, 29.3% (*n* = 61) were Senior Firefighters, 27.9% (*n* = 58) were Fire Sergeants, and 20.2% (*n* = 42) were Fire Lieutenants or had higher ranks. The three-shift work pattern was the most common (79.3%, *n* = 165). The mean for years of continuous service was 9.18 ± 6.76, and the mean frequency of mobilizations per week was 6.49 ± 3.38. The mean monthly salary was 3,677,900 ± 726,100 KRW. The most common license held was that of registered nurse (44.2%, *n* = 92), and 78.4% (*n* = 163) reported an intention to stay (Table 1).

Burnout significantly differed by sex (t = −2.63, *p* = 0.009), licenses (F = 6.51, *p* < 0.001), frequency of mobilization (F = 9.90, *p* < 0.001), and intention to stay (t = −5.38, *p* < 0.001). Burnout levels were significantly higher among female participants and among those who reported no intention to stay. Scheffé’s post hoc analysis showed that burnout was highest among those holding a registered nurse license and among those with a frequency of mobilization of ≥5 times per week.

### 3.2. Descriptive Statistics and Correlations Among Study Variables

Table 2 presents the descriptive statistics and correlations among the variables. The mean score for WPV was 1.61 ± 0.76 (range 1–5), PTS was 0.82 ± 0.84 (range 0–4), resilience was 2.50 ± 0.75 (range 0–4), and burnout was 2.40 ± 1.07 (range 0–6). Burnout was positively correlated with WPV (r = 0.33, *p* < 0.001) and PTS (r = 0.58, *p* < 0.001) but negatively correlated with resilience (r = −0.30, *p* < 0.001).

### 3.3. Model Fit

Prior to conducting path analysis, multicollinearity and autocorrelation among the variables were assessed. The absolute values of the correlations between variables ranged from 0.20 to 0.58. The absolute values of skewness ranged from 0.01 to 1.53 and were all below 2. The absolute values of kurtosis ranged from 0.39 to 2.39 and were all below 4. Residual analyses confirmed normality, independence, and homoscedasticity. The variance inflation factor (VIF) values ranged from 1.30 to 5.07 and were all less than 10, indicating no multicollinearity among the independent variables. The Durbin–Watson statistic was 2.15, confirming the absence of autocorrelation in the dependent variable and verifying that the assumptions for path analysis were met.

The model was estimated using the maximum likelihood method, and the resulting fit indices were as follows: χ^2^ = 6.49 (df = 5), GFI = 0.991, CFI = 0.995, NFI = 0.979, IFI = 0.995, TLI = 0.979, SRMR = 0.014, and RMSEA = 0.038. All indices met the recommended cutoff criteria, supporting the appropriateness of the final model. Table 3 presents the model fit results.

### 3.4. Path Analysis

**Figure 1:**
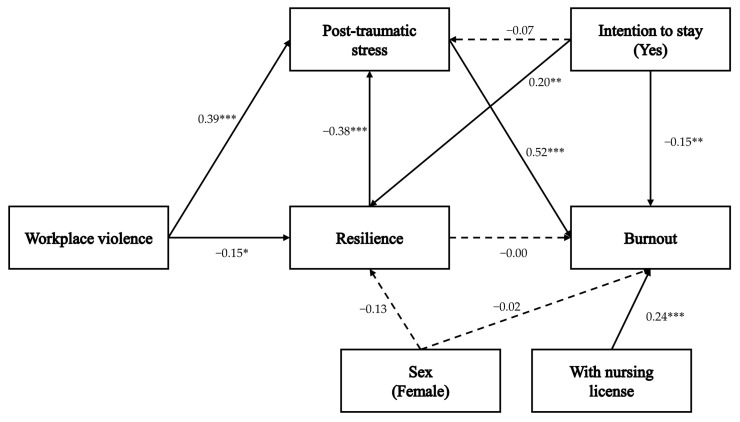
A path diagram of the study. *: *p* < 0.05, **: *p* < 0.01, ***: *p* < 0.001.

The path analysis results are presented in Table 4, and the corresponding model is illustrated in Figure 1 (see also Supplementary Figures S1–S3).

For resilience, WPV (β = −0.15, *p* = 0.025, 95% CI [−0.29, −0.02]) and intention to stay (β = 0.20, *p* = 0.009, 95% CI [0.06, 0.33]) showed statistically significant associations, accounting for 10.6% of this variable’s variance. Gender did not have a significant direct effect on resilience (β = −0.13, *p* = 0.062, 95% CI [−0.25, −0.01]).

For PTS, WPV (β = 0.39, *p* = 0.001, 95% CI [0.28, 0.49]) and resilience (β = −0.38, *p* = 0.001, 95% CI [−0.48, −0.26]) had significant direct effects, explaining 39.1% of the variance. Intention to stay (β = −0.07, *p* = 0.188, 95% CI [−0.19, 0.03]) did not have a significant direct effect; however, indirect effects were significant for intention to stay (β = −0.08, *p* = 0.008, 95% CI [−0.14, −0.02]) through resilience as well as for WPV (β = 0.06, *p* = 0.021, 95% CI [0.01, 0.12]).

For burnout, PTS (β = 0.52, *p* = 0.001, 95% CI [0.40, 0.62]), possession of a nursing license (β = 0.24, *p* = 0.001, 95% CI [0.14, 0.35]), and intention to stay (β = −0.15, *p* = 0.009, 95% CI [−0.26, −0.05]) showed statistically significant associations, explaining 44.0% of the variance. Resilience (β = 0.00, *p* = 0.960, 95% CI [−0.12, 0.11]) and gender (β = −0.02, *p* = 0.791, 95% CI [−0.13, 0.10]) had no significant direct effects. However, indirect effects on burnout were observed for WPV (β = 0.23, *p* = 0.001, 95% CI [−0.16, 0.31]), resilience (β = −0.20, *p* = 0.002, 95% CI [−0.31, −0.08]), gender (β = 0.03, *p* = 0.037, 95% CI [0.00, 0.07]), and intention to stay (β = −0.08, *p* = 0.012, 95% CI [−0.15, −0.02]) through PTS.

## 4. Discussion

This study’s path model demonstrated an overall good fit, with most indices meeting the recommended thresholds. The model test revealed that WPV did not have a significant direct effect on burnout; however, its indirect effect through PTS was significant, indicating that the association between the two variables was only indirect through PTS. Resilience did not have a significant direct effect on burnout, but its indirect effect on PTS was significant, indicating a negative influence on burnout. Among all of the variables, PTS showed the strongest association with burnout. Gender, type of professional license, and intention to stay were identified as significant general characteristics of burnout. Based on these results, the relationships among the variables were examined, and their practical applications and policy implications were discussed.

In this study, PTS was identified as the most influential factor contributing to burnout among paramedics. However, few studies have examined the impact of PTS on burnout specifically among paramedics. In contrast, studies on firefighters have reported PTS as a significant predictor of burnout but not as the most influential factor [30,31]. Therefore, the significance of this study lies in demonstrating that PTS exerts a particularly strong effect on burnout among paramedics, underscoring the importance of managing PTS for the prevention and control of burnout in this group.

In this study, WPV among paramedics did not affect burnout directly but influenced it only through PTS. A previous study of paramedics [32] reported that the frequency of exposure to critical incidents, including WPV, did not affect burnout, showing that the frequency of critical incident exposure alone was insufficient to explain individual burnout levels. In contrast, this study statistically verified an indirect-only pathway, showing that exposure to critical incidents was not directly associated with burnout but was indirectly associated with trauma responses, namely, PTS. Furthermore, a meta-analysis of nurses’ experiences of WPV [33] reported that such experiences more than doubled the risk of both PTSD and burnout but did not verify PTS as a link between WPV and burnout. This study fills this gap by demonstrating an indirect pathway whereby WPV is associated with burnout through PTS, suggesting that, in practice, PTSD screening and intervention following WPV are essential strategies for reducing the risk of burnout.

Additionally, this study found that WPV experienced by paramedics had a negative effect on resilience. This suggests that repeated WPV can trigger psychological trauma responses, deplete resources, and eventually lead to burnout. These results can be explained by the conservation of resources (COR) theory. According to the COR theory, individuals are motivated to preserve personal resources such as emotional stability, self-esteem, and a sense of control, and even non-physical violence (e.g., insults, blame) acts as a resource-loss event [34]. The repeated loss of resources results in emotional exhaustion and depersonalization, which can exacerbate burnout. Therefore, in practice, it is essential to implement interventions that not only reduce exposure to WPV but also actively support the recovery of psychological resources.

This study confirmed that resilience does not serve as a protective factor against burnout. This finding contrasts with prior studies [35] reporting resilience as a key buffering resource against burnout, indicating the need for further research on the role of resilience among paramedics. Recent studies have highlighted that resilience may not operate independently; instead, it may exert protective effects through interactions with emotional and social resources. For example, coworker support has been shown to mitigate trauma responses such as PTSD and facilitate recovery [15], and greater coworker support is associated with a reduced risk of psychopathology following WPV [36]. In addition, resilience increases as the levels of coworker support and self-encouragement increase, with coworker support reported to have a greater influence [37]. Similarly, a prior study on firefighters and paramedics [36] identified perceived coworker support as a key moderator in the relationship between WPV and PTSD and the risk of psychopathology. Notably, high levels of coworker support produced a buffering effect, substantially reducing the negative impact of WPV on mental health, while personal support resources, such as family, failed to provide comparable protection. Thus, future research should focus on the indirect protective mechanisms of resilience, particularly its interaction with coworker support. The absence of a protective role for resilience in this study may be attributed to both the limitations of the cross-sectional design, which restrict causal interpretation, and the potential for a “saturation effect,” whereby resilience’s impact is diminished under conditions of severe or repeated violence exposure. This possibility warrants further investigation in future studies. In addition, future studies should examine whether resilience mediates or moderates the relationship between WPV and PTS and attempt to develop coworker support-enhancement programs for resilience promotion.

This study went beyond conducting a path analysis of the main variables to identify groups vulnerable to burnout by incorporating general characteristics. The results showed that sex, type of license, and intention to stay emerged as significant predictors of burnout, with females, individuals holding nursing licenses, and those with lower intentions to stay demonstrating relatively higher burnout levels. Notably, holding a nursing license was the second strongest predictor of burnout among paramedics after PTS, suggesting that the psychological burden associated with professional roles may substantially contribute to burnout. Glawing et al. (2023) [38] highlighted that the responsibility of making independent clinical decisions as the only nurse in an ambulance constituted a major stressor, and the interview comment “The greatest stress was that there was no other nurse with me” further illustrated how expectations for solo nursing roles in emergency situations may lead to considerable psychological strain. Furthermore, the intention to stay may have operated not only as a willingness to continue in the profession but also as a psychological strategy for cognitive reappraisal or emotional regulation under stressful conditions, which can be understood as a defense mechanism in stress appraisal and coping processes. Because this study employed a cross-sectional design, the observed association between intention to stay and burnout should not be interpreted as a causal relationship. This functional interpretation requires empirical validation in future studies. Therefore, interventions should incorporate tailored emotional support that reflects differences in sex and professional roles among paramedics, along with strategies aimed at reinforcing their intention to stay. These findings hold significant academic and practical value by providing scientific evidence to guide high-risk group screening, design of tailored interventions, and prioritization of resource allocation.

This study has significant academic and practical value as it went beyond simply enumerating factors of burnout among paramedics to reveal the interactions of complex variables through path analysis. In contrast to prior research, this study identified both the absence of resilience as a protective factor and the influence of general characteristics (sex, licenses, and intention to stay) on burnout. However, it has several limitations. First, the cross-sectional design limits the ability to clearly interpret causal relationships among variables. Second, because the sample consisted of only paramedics belonging to a single metropolitan city (Busan), the generalizability of the results is limited. Third, as all variables were measured using self-reported questionnaires, the possibility of response bias cannot be ruled out. Finally, because this was a cross-sectional study, the temporal order and causal directions among burnout, intention to stay, and other predictors cannot be established. Therefore, the path analysis estimates should be interpreted as statistical associations within a hypothesized model rather than proven causal effects. Reverse causality is also possible (e.g., higher burnout may reduce the intention to stay). Future longitudinal studies are warranted to clarify these relationships.

Future research should pursue cohort studies that monitor changes in trauma responses and burnout over time following violent incidents, qualitative investigations of recovery processes through in-depth interviews, and randomized controlled trials (RCTs) to evaluate the effectiveness of WPV response training and debriefing programs. These studies would provide empirical evidence to safeguard the mental health of paramedics and support their recovery.

## 5. Conclusions

This study developed a model to investigate how PTS, WPV, and resilience influence burnout among paramedics, and it analyzed the pathways and magnitudes of these relationships. The findings revealed that WPV was directly associated with both PTS and resilience and that PTS was directly associated with burnout. Although resilience did not directly influence burnout, it did indirectly contribute to burnout through PTS. Furthermore, the results indicated that being female, being a nurse holding a license, and having a lower intention to stay showed significant associations with burnout.

Drawing on these results, strategies to prevent burnout among paramedics have been proposed at the individual, team, organizational, and national levels. At the individual level, early screening for PTSD should be implemented, and evidence-based recovery interventions, such as Prolonged Exposure (PE) and Eye Movement Desensitization and Reprocessing (EMDR) should be used to prevent the chronicity of trauma responses and strengthen recovery resources. At the team level, group resilience can be promoted through workshops and emotional support training, which enhances resilience and coworker support. At the organizational level, institutionalized systems should be established, including zero-tolerance policies for violence, measures to ensure psychological safety, and mechanisms for reporting and post-incident support. At the national level, penalties for violence against paramedics should be reinforced, and standardized intervention models, along with financial support for mental health recovery, should be established. These multilevel approaches will facilitate timely assessment and intervention for PTS following WPV, mitigate burnout, and ultimately provide a foundation for sustaining an EMS and ensuring the quality of its services.

## Figures and Tables

**Table 1:** General Characteristics and Differences in Burnout according to General Characteristics in Participants (*n* = 208).

Variable	Characteristics	n (%)	M ± SD (Range)	Burnout		
				M ± SD	t/F	*p* (Scheffe)
Sex	Male	167 (80.3)		2.30 ± 1.08	−2.63	0.009
	Female	41 (19.7)		2.79 ± 0.94		
Age (year)	≤30	31 (14.9)	37.51 ± 7.12	2.34 ± 1.15	0.09	0.964
	31~35	70 (33.7)	(23~59)	2.41 ± 1.05		
	36~40	40 (19.2)		2.47 ± 0.98		
	≥41	67 (32.2)		2.38 ± 1.12		
Marital status	Single/Divorced/Widowed	64 (30.8)		2.35 ± 1.14	−0.43	0.665
	Married	144 (69.2)		2.42 ± 1.04		
Position	Firefighter	47 (22.6)		2.41 ± 1.00	1.96	0.121
	senior Firefighter	61 (29.3)		2.42 ± 1.10		
	Fire Sergeant	58 (27.9)		2.60 ± 1.00		
	Fire Lieutenant or higher	42 (20.2)		2.08 ± 1.15		
Shiftwork	two-shift	28 (13.5)		2.61 ± 1.04	0.73	0.481
	three-shift	165 (79.3)		2.36 ± 1.05		
	regular day shift	15 (7.2)		2.48 ± 1.29		
licenses	emergency medical technician ^a^	57 (27.4)		2.10 ± 0.96	6.51	<0.001
	nurse ^b^	92 (44.2)		2.75 ± 1.01		a, c, d < b
	completion of training on emergency medical service ^c^	44 (21.2)		2.14 ± 1.03		
	none ^d^	15 (7.2)		2.13 ± 1.36		
Years of	<5	66 (31.7)	9.18 ± 6.76	2.48 ± 1.08	0.31	0.734
continuous service	5~10	63 (30.3)	(0.25~33.50)	2.33 ± 1.02		
	≥10	79 (38.0)		2.39 ± 1.10		
Frequency of	<5 times ^a^	57 (27.4)	6.49 ± 3.38	1.90 ± 1.08	9.90	<0.001
mobilization	5~10 times ^b^	93 (44.7)	(0~14)	2.53 ± 0.94		a < b, c
(per week)	≥10 times ^c^	58 (27.9)		2.69 ± 1.10		
monthly salary	<350	85 (40.9)	367.79 ± 72.61	2.42 ± 1.03	0.23	0.792
(10,000 won)	350~400	50 (24.0)	(200~600)	2.46 ± 1.12		
	≥400	73 (35.1)		2.33 ± 1.08		
intention to stay	Yes	163 (78.4)		2.20 ± 1.01	−5.38	<0.001
	No	45 (21.6)		3.11 ± 0.98		

Note: Superscript letters (a–d) denote post-hoc groupings within each comparison; values that share a letter are not significantly different, whereas values with different letters differ significantly (Scheffé’s post-hoc test, *p* < 0.05). Letters are assigned independently within each variable and do not represent the same group across different variables.

**Table 2:** Level of Study Variables and Pearson’s correlations of Related Variables.

Variables	Mean ± SD	Skewness	Kurtosis	Workplace Violence	Resilience	Post-Traumatic Stress
				r(p)		
Workplace violence (range 1–5)	1.61 ± 0.76	0.60	2.12			
Resilience (range 0–4)	2.50 ± 0.75	−0.01	−0.48	−0.20 (0.005)		
Post-traumatic stress (range 0–4)	0.82 ± 0.84	1.53	2.39	0.49 (<0.001)	−0.47 (<0.001)	
Burnout (range 0–6)	2.40 ± 1.07	0.19	0.39	0.33 (<0.001)	−0.30 (<0.001)	0.58 (<0.001)

**Table 3:** Goodness of fit.

	χ^2^	df	GFI	CFI	NFI	IFI	TLI	SRMR	RMSEA
Model	6.49	5	0.991	0.995	0.979	0.995	0.979	0.014	0.038
Criterion	**χ**^2^/df < 3		≥0.9	≥0.9	≥0.9	≥0.9	≥0.9	<0.05	<0.05

**Table 4:** Path coefficients of final model.

Endogenous Variables	Exogenous Variables	Hypothetical Model				Direct Effect		Indirect Effect		Total Effect		SMC
		β	SE	CR	*p*	β (*p*)	95% CI	β (*p*)	95% CI	β (*p*)	95% CI	
Resilience	Workplace violence	−0.15	0.07	−2.17	0.030	−0.15 (0.025)	−0.29~−0.02			−0.15 (0.025)	−0.29~−0.02	0.106
	Sex (female)	−0.13	0.13	−1.87	0.062	−0.13 (0.062)	−0.25~0.01			−0.13 (0.062)	−0.25~0.01	
	Intention to stay (Yes)	0.20	0.13	2.79	0.005	0.20 (0.009)	0.06~0.33			0.20 (0.009)	0.06~0.33	
Post-traumatic stress	Workplace violence	0.39	0.06	6.87	<0.001	0.39 (0.001)	0.28~0.49	0.06 (0.021)	0.01~0.12	0.45 (0.001)	0.34~0.55	0.391
	Resilience	−0.38	0.06	−6.63	<0.001	−0.38 (0.001)	−0.48~−0.26			−0.38 (0.001)	−0.48~−0.26	
	Sex (female)							0.05 (0.057)	0.00~0.10	0.05 (0.057)	0.00~0.10	
	Intention to stay (Yes)	−0.07	0.12	−1.27	0.206	−0.07 (0.188)	−0.19~0.03	−0.08 (0.008)	−0.14~−0.02	−0.15 (0.012)	−0.27~−0.03	
Burnout	Workplace violence							0.23 (0.001)	0.16~0.31	0.23 (0.001)	0.16~0.31	0.440
	Resilience	0.00	0.09	−0.03	0.979	0.00 (0.960)	−0.12~0.11	−0.20 (0.001)	−0.27~−0.13	−0.20 (0.002)	−0.31~−0.08	
	Post-traumatic stress	0.52	0.08	8.63	<0.001	0.52 (0.001)	0.40~0.62			0.52 (0.001)	0.40~0.62	
	Sex (female)	−0.02	0.16	−0.25	0.800	−0.02 (0.791)	−0.13~0.10	0.03 (0.037)	0.00~0.07	0.01 (0.873)	−0.10~0.13	
	With nursing licenses	0.24	0.12	4.18	<0.001	0.24 (0.001)	0.14~0.35			0.24 (0.001)	0.14~0.35	
	Intention to stay (Yes)	−0.15	0.15	−2.63	0.009	−0.15 (0.009)	−0.26~−0.04	−0.08 (0.012)	−0.15~−0.02	−0.23 (0.001)	−0.35~−0.10	

## Data Availability

The datasets generated and/or analyzed during the current study are not publicly available due to privacy and ethical restrictions. De-identified data may, however, be made available from the corresponding author on reasonable request and with Institutional Review Board approval.

## Supplementary Materials

The following supporting information can be downloaded at: https://www.mdpi.com/article/10.3390/healthcare13192519/s1, Figure S1. Resilience-related pathways. Figure S2. Post-traumatic stress-related pathways. Figure S3. Burnout-related pathways.
