# Supplementary material for: Post-Traumatic Stress, Workplace Violence, Resilience, and Burnout: A Path Analysis Among Korean Paramedics
Source: Healthcare (Basel). 2025 Oct 4;13(19):2519. doi: 10.3390/healthcare13192519 (PMC12524421; doi:10.3390/healthcare13192519)

Figure S1. Resilience-related pathways. *: $p<0.05$, ***: $p<0.001$. Workplace violence had a significant negative effect on resilience, and resilience in turn significantly reduced post-traumatic stress. The effect of resilience on burnout was significant only indirectly through PTSD (β _indirect ≈ −0.20). For clarity, only significant direct effects are displayed; indirect effects are described in Table 4.

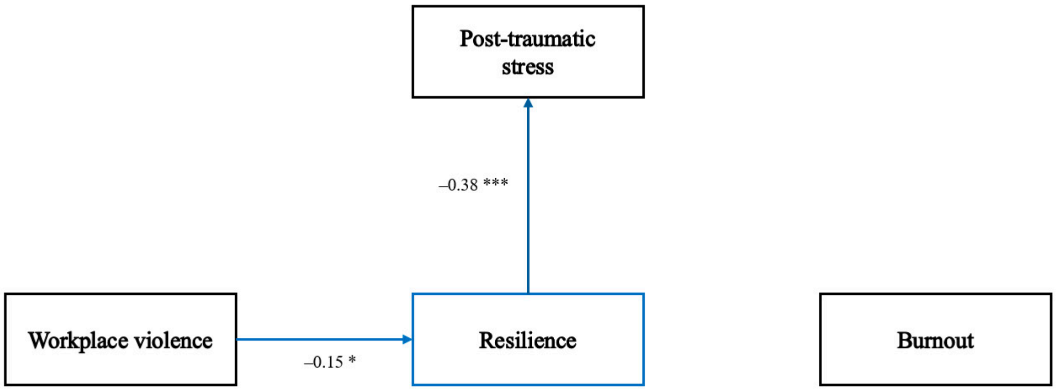

Figure S2. Post-traumatic stress-related pathways. ***: $p<0.001$. Workplace violence had a significant positive effect on post-traumatic stress, whereas resilience significantly reduced post-traumatic stress. Post-traumatic stress, in turn, significantly increased burnout. Indirect effects, such as workplace violence and resilience influencing burnout through post-traumatic stress, were also significant. For clarity, only significant direct effects are displayed; indirect effects are described in Table 4.

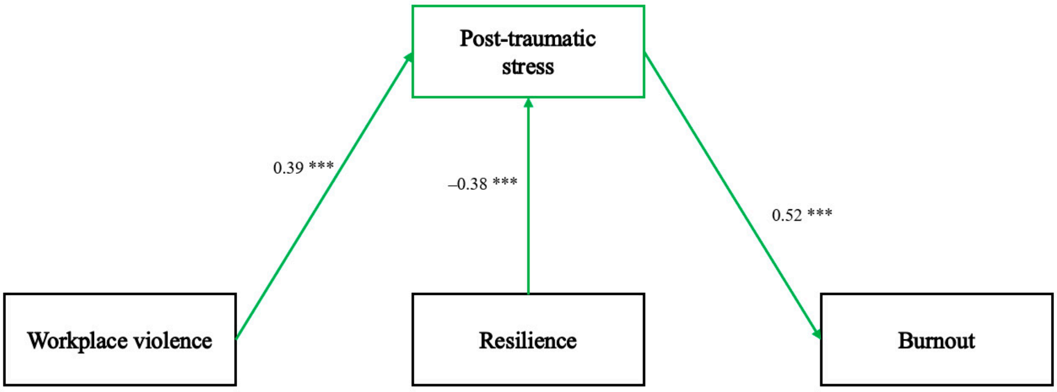

Figure S3. Burnout-related pathways. **: p<0.01, ***: p<0.001 . Post-traumatic stress had a significant positive effect on burnout, whereas intention to stay showed a significant negative effect and holding a nursing license showed a significant positive effect. In addition, workplace violence, resilience, and sex influenced burnout only indirectly through post-traumatic stress. For clarity, only significant direct effects are displayed; indirect effects are described in Table 4.

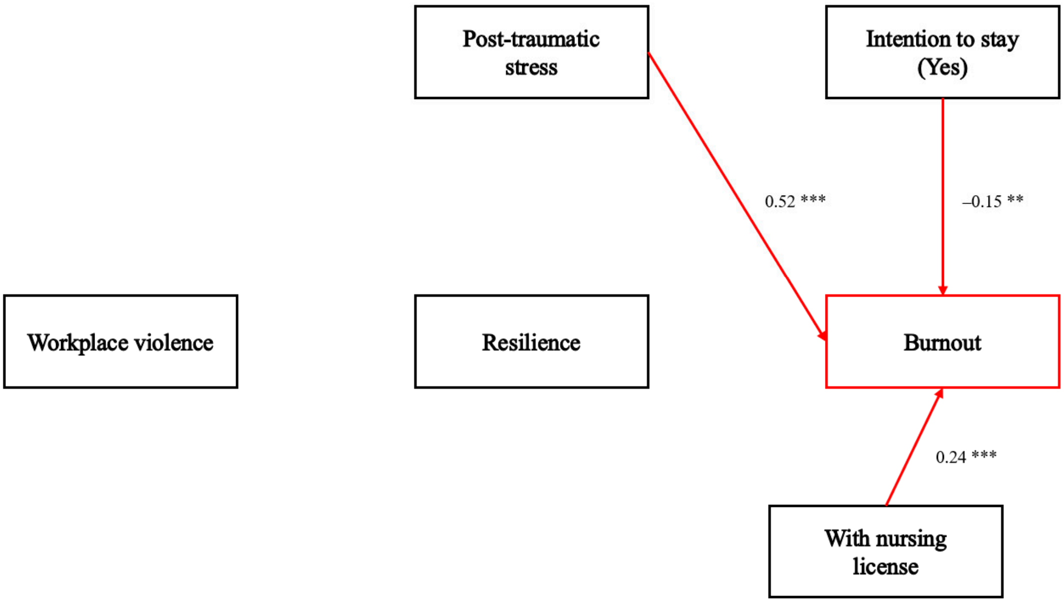

Supplement: Supplementary file 1 [file healthcare-13-02519-s001.zip › healthcare-3874182-supplementary.pdf]
